# *QuickStats:* Age-Adjusted Death Rates* from Diabetes Mellitus^†^ Among Adults Aged ≥65 Years, by Single Race and Hispanic Origin — National Vital Statistics System, United States, 2020

**DOI:** 10.15585/mmwr.mm7131a5

**Published:** 2022-08-05

**Figure:**
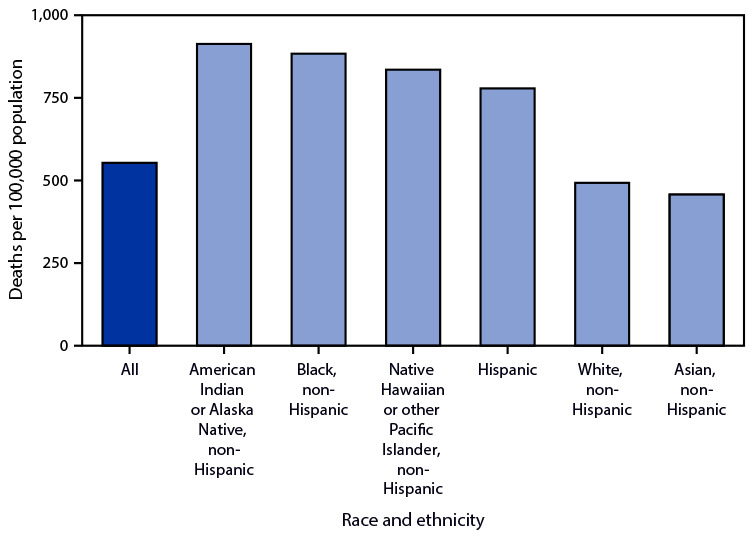
In 2020, the age-adjusted death rate from diabetes mellitus as the underlying or contributing cause of death for adults aged ≥65 years was 553.4 per 100,000 population. The rates were higher for non-Hispanic American Indian or Alaska Native (913.6), non-Hispanic Black (884.1), non-Hispanic Native Hawaiian or other Pacific Islander (835.4), and Hispanic adults (778.5) compared with non-Hispanic White (493.3) and non-Hispanic Asian adults (457.7). Rates were lower among Hispanic than among non-Hispanic American Indian or Alaska Native and non-Hispanic Black adults, and rates were lower among non-Hispanic Asian compared with non-Hispanic White adults.

**Source:** National Vital Statistics System, 2020. https://www.cdc.gov/nchs/nvss/deaths.htm

